# Identification of Key Regulators of Hepatitis C Virus-Induced Hepatocellular Carcinoma by Integrating Whole-Genome and Transcriptome Sequencing Data

**DOI:** 10.3389/fgene.2021.741608

**Published:** 2021-09-09

**Authors:** Guolin Chen, Wei Zhang, Yiran Ben

**Affiliations:** Department of Infectious Diseases, The First Affiliated Hospital of Harbin Medical University, Harbin, China

**Keywords:** genetic mutation, transcriptome, miRNA, hepatitis C. virus, hepatocellular carcinoma

## Abstract

**Background:** Hepatitis C virus (HCV) infection is a major cause of cirrhosis and hepatocellular carcinoma (HCC). Despite recent advances in the understanding of the biological basis of HCC development, the molecular mechanisms underlying HCV-induced HCC (HCC-HCV) remain unclear. The carcinogenic potential of HCV varies according to the genotype and mutation in its viral sequence. Moreover, regulatory pathways play important roles in many pathogenic processes. Therefore, identifying the pathways by which HCV induces HCC may enable improved HCC diagnosis and treatment.

**Methods:** We employed a systematic approach to identify an important regulatory module in the process of HCV-HCC development to find the important regulators. First, an HCV-related HCC subnetwork was constructed based on the gene expression in HCC-HCV patients and HCC patients. A priority algorithm was then used to extract the module from the subnetworks, and all the regulatory relationships of the core genes of the network were extracted. Integrating the significantly highly mutated genes involved in the HCC-HCV patients, core regulatory modules and key regulators related to disease prognosis and progression were identified.

**Result:** The key regulatory genes including *EXO1*, *VCAN*, *KIT*, and *hsa-miR-200c-5p* were found to play vital roles in HCV-HCC development. Based on the statistics analysis, *EXO1*, *VCAN*, and *KIT* mutations are potential biomarkers for HCV–HCC prognosis at the genomic level, whereas has-miR-200c-5P is a potential biomarker for HCV–HCC prognosis at the expression level.

**Conclusion:** We identified three significantly mutated genes and one differentially expressed miRNA, all related to HCC prognosis. As potential pathogenic factors of HCC, these genes and the miRNA could be new biomarkers for HCV-HCC diagnosis.

## Introduction

Hepatocellular carcinoma (HCC)—the second leading cause of cancer−related deaths worldwide (Merte, 1989)—is often diagnosed at an advanced stage and progresses rapidly. Therefore, in HCC patients, early diagnosis is very important to improve their prognosis. Currently, early clinical screening methods for HCC involve serum alpha fetoprotein (AFP) detection and liver ultrasound examination (Sato et al., 1993). However, the sensitivity and specificity of markers such as AFP are marginal; moreover, ultrasound examination considerably relies on the subjective judgment of the operator, and conventional ultrasound results are often not useful for the conclusive identification of liver lesions. Therefore, a more effective, accurate method for screening liver cancer is needed urgently. As the understanding of cancer biology improves, liquid biopsy will become an increasingly useful tool for early diagnosis. Risk factors for HCC include cirrhosis, aflatoxin B intake, alcohol consumption, and hepatitis B virus (HBV) and hepatitis C virus (HCV) infection. Of these, HBV and HCV infections are the most notorious; in general, HBV- or HCV-positive patients have a 15–20-fold higher lifetime relative risk of HCC than HBV- and HCV-negative patients (El-Serag, 2012). To date, few studies have been focused on the factors leading to liver cancer in HCV patients. At present, HCV RNA, cirrhosis, and HCV genotype are thought to affect the occurrence of HCV-related liver cancer, but the involvement of these factors has not been conclusively proven. At present, the number of people affected by chronic HCV infection is 180 million—linked to > 350,000 deaths annually (Li and Lo, 2015). Epidemiological studies have also shown that HCV is a risk factor for various diseases, including oral manifestations, glomerulopathies, type 2 diabetes mellitus, and insulin resistance (Montenegro et al., 2013; Carrozzo and Scally, 2014; Ozkok and Yildiz, 2014).

In total, 55–85% of people with HCV infection will develop chronic hepatitis C, and 20–30% of people with chronic liver disease will develop liver failure or cirrhosis (Mahale et al., 2017). Over the course of 30 years, 1–3% of patients with HCV without cirrhosis will develop HCC eventually (Huang et al., 2011; El-Serag, 2012). Moreover, one-third of HCC cases have been reported to be caused by hepatitis C (Parkin, 2006). At present, there are three major known mechanisms for HCV-induced HCC (HCV-HCC): direct pathways involving HCV core proteins, indirect pathways caused by oxidative stress and steatosis, and microRNA (miRNA)-related pathways (Tholey and Ahn, 2015). While biological signaling systems are complex, the analysis of linear pathways may still provide valuable insights (Weng et al., 1999). In the study of HCV, core genes have been found to be closely related to the carcinogenicity of chronic HCV infection. The expression of core genes has been experimentally shown to immortalize primary liver cells and induce cell transformation and carcinogenesis (Li et al., 2010). In addition, the genome sequencing analysis has demonstrated significant differences in the characteristics of liver cancer patients with or without HCV (Fishman et al., 2009). Taken together, these results indicate that core HCV gene mutations are closely associated with increased liver cancer risks.

In this study, the correlation between the key regulators and prognosis was investigated by integrating whole-genome and transcriptome sequencing data from The Cancer Genome Atlas (TCGA) and Gene Expression Omnibus (GEO) databases. We identified differentially expressed and mutated genes between HCV-HCC and HCC groups and performed functional enrichment analysis for genes in the module. Then, we explored the association of the key regulators with patient prognoses. The module and the key regulators may be potential biomarkers for predicting HCV-HCC.

## Materials and Methods

### The Cancer Genome Atlas and Gene Expression Omnibus Data Acquisition

Gene mutation and mRNA and miRNA expression data as well as clinical information were downloaded from TCGA^1^ (Deng et al., 2016). In TCGA, liver hepatocellular carcinoma (LIHC) samples are divided into two groups: the first group contains HCV RNA or genotype or hepatitis C antibody in the patient’s clinical information, and the other group does not; here, we named the two groups HCC-HCV and HCC. The gene/miRNA microarray as verifying cohorts GSE154211 (Wang et al., 2021) and GSE119159 (Umezu et al., 2020) were downloaded from GEO database. The data were normalized, and R and its packages were employed in all analysis steps.

### Differential Analysis

MuTect2 Somatic Mutation data, analyzed using MuTect2, were download from TCGA. TCGA provides somatic mutation data in the MAF format. Therefore, we visualized somatic mutations using the R package “maftools” (Mayakonda et al., 2018). In total, 96 HCC-HCV samples and 269 HCC samples were present in the dataset. We calculated the mutational status of genes using the algorithm in maftools, and the genes with *p* < 0.05, OR > 2, and number of mutations > 5 were selected as the significantly and differentially mutated genes.

According to the groupings, we performed normalization and differential gene expression analysis using the R package “edgeR.” False discovery rate (FDR) < 0.01 and | log_2_ fold change (FC)| > 1 were used as cutoffs for identify differentially expressed genes (DEGs) for further analysis. Two R packages “pheatmap” and “ggplot2” were used for visualizing the heatmaps and volcano maps, respectively.

In total, 139 HCV patients were enrolled in the GSE119159. They included 99 patients who had not developed HCC and 40 who had developed HCC. A total of 10 samples (tumor and non-tumor regions) from two HCV-related HCC patients and three HCC patients were used to find the gene candidates in HCV-related HCC in the GSE154211. For differential expression analysis, we used the R package “limma.” | log2FC| > 1 and logFDR < 0.01 were used as cutoffs to identify DEGs for further analysis.

### Construction of the Transcription Factor–miRNA–mRNA Regulatory Network

The human transcription factor (TF) and miRNA regulatory networks were constructed by integrating miRTarBase, TRANSFAC, and TransmiR (Vlachos et al., 2015; Chou et al., 2018; Tong et al., 2019). The three databases include curated interactions among human TFs, miRNAs, and target genes. We uniformly named the genes and miRNAs within the regulatory networks according to the National Center for Biotechnology Information (NCBI) and miRbase databases. Moreover, all regulatory relationships within the regulatory network were supported experimentally. In total, 888 TFs, 1,072 miRNAs, 3,150 target genes, and 18,056 edges were discovered in the regulatory network.

### Functional Enrichment Analysis

The key regulatory gene symbols were converted to Entrez ID using the R package “org.Hs.eg.db.” To identify the biological pathways involved in HCV-HCC occurrence and development, we employed Gene Ontology–biological process (GO-BP) function and Kyoto Encyclopedia of Genes and Genomes (KEGG) pathway enrichment analysis and visualized the results using the R packages “clusterProfiler” and “ggplot2.”

### Survival Analysis

We constructed an HCV-HCC-related subnetwork and identified key regulators from the subnetworks. Next, we investigated whether the key regulators could distinguish HCC patients with good or poor outcomes. From these data, we obtained TCGA HCC dataset with mRNA/miRNA expression and clinical information. Then, we used the key regulator expression values and mutation information to cluster all patients into two groups. The differential survival of the two study groups was finally assessed using the log-rank test.

## Results

### Mutation Analysis

**FIGURE 1:**
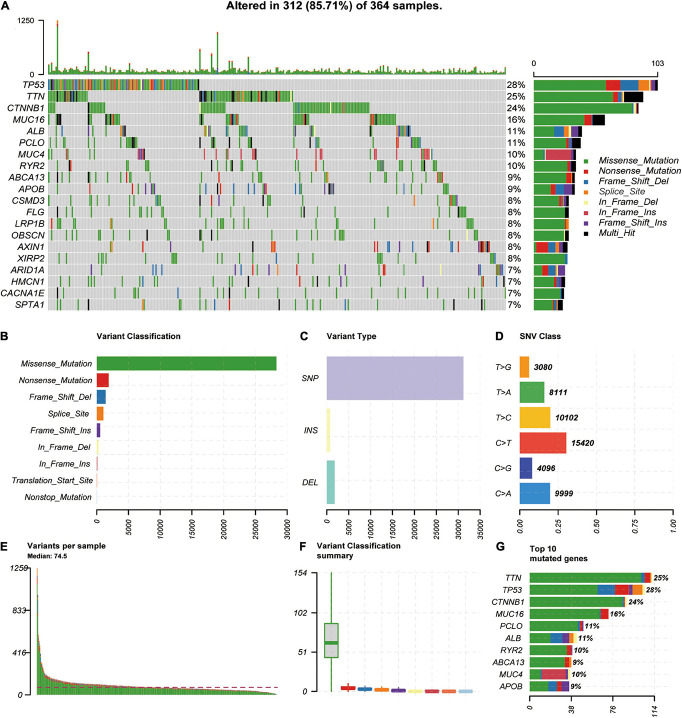
Genome–wide mutation profiles in LIHC. **(A)** Landscape of mutation profiles in LIHC samples. Mutation information of each gene is shown in the waterfall plot, where different colors represent different types of variation. **(B–D)** Cohort summary plot displays distribution of variants according to variant classification, type, and SNV class. **(E)** Mutation load in each sample. **(F)** Variant classification in each sample. **(G)** Top 10 mutated genes in LIHC. LIHC, liver hepatocellular carcinoma; SNV, single-nucleotide variant.

**FIGURE 2:**
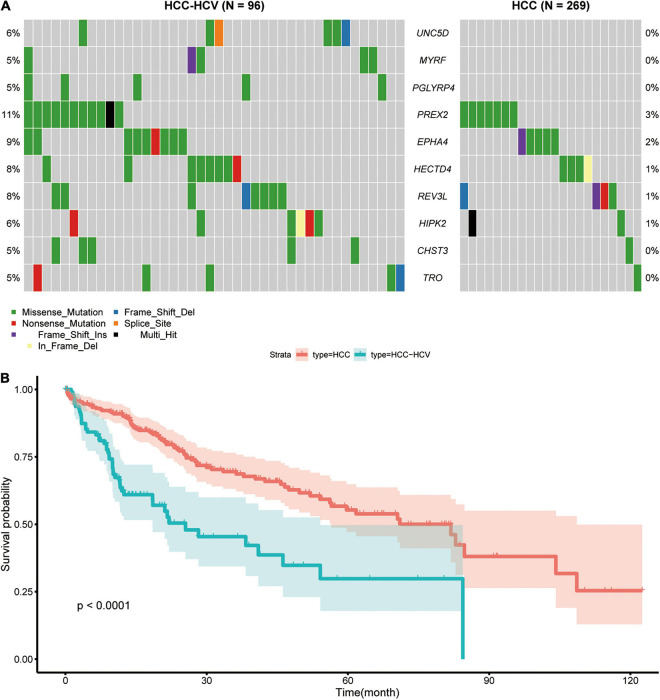
Analyses of different somatic mutations and survival time in HCC-HCV and HCC samples. **(A)** Waterfall plot of detailed information of top 10 differentially mutated genes in each group. **(B)** K-M curves of patients in the HCC group and HCC-HCV group. HCC, hepatocellular carcinoma; HCV, hepatitis C virus; K-M, Kaplan–Meier.

We downloaded and analyzed the somatic mutation data of 392 TCGA-LIHC samples. The mutation information of all genes in the samples is displayed as a waterfall diagram, with different colors representing different mutation types (Figure 1A). Further analysis showed that missense mutation, single-nucleotide polymorphisms (SNPs), and C > T accounted for the highest proportion of the variations (Figures 1B–D). The median number of variations in all HCC samples was 74.5, and the maximum number of variations in a single sample was 1,250 (Figure 1E). The number of variations in different classifications in all samples is shown in a box diagram (Figure 1F). The top 10 mutated genes in the 392 samples were TTN (25%), TP53 (28%), CTNNB1 (24%), MUC16 (16%), PCLO (11%), ALB (11%), RYR2 (10%), ABCA13 (9%), MUC4 (10%), and APOB (9%; Figure 1G). In total, 96 HCC-HCV samples and 269 HCC samples were present in TCGA dataset; the survival analysis indicated that HCC patients without HCV lived significantly longer than HCC-HCV patients (Figure 2B). With the use of the maftools algorithm, 41 differentially mutated genes were identified (Supplementary Table 1). The top 10 differential mutated genes between the two groups of patients were UNC5D (6–0), MYRF (5–0), PGLYRP4 (5–0), PREX2 (11–7), EPHA4 (9–5), HECTD4 (8–4), REV3L (8–4), HIPK2 (6–2), CHST3 (5–1), and TRO (5–1; Figure 2A). Moreover, HCC-HCV patients with mutations in some genes had a poor prognosis (Supplementary Figure 1).

### Transcriptome Analysis

Differentially expressed mRNAs and miRNAs were identified from two raw datasets: one containing GSE154211 and GSE119159 downloaded from the GEO database and another dataset from TCGA database. In total, 530 mRNA and 30 miRNA transcripts were observed to be expressed differentially in the HCC-HCV samples compared with HCC samples in TCGA dataset—including, respectively, 412 and 25 upregulated and 118 and five downregulated transcripts. Hierarchical clustering showed systematic variations in mRNA and miRNA expression in the HCC-HCV and HCC samples (Supplementary Figure 2). To identify the genes related to HCC-HCV in GSE154211, we first divided the expression data into four groups to identify DEGs between (A) HCC vs. HCC-HCV-adjacent, suggesting related to HCV-related carcinogenesis; (B) HCC-HCV vs. HCC, suggesting related HCV-related hepatocarcinogenesis; (C) HCC-HCV-adjacent vs. HCC-adjacent, suggesting related to HCV-related non-oncogenic effects; and (D) HCC vs. HCC-adjacent, suggesting related to non-HCV-related carcinogenesis. Four groups of data were then analyzed. Consequently, we identified 1,494 DEGs belonging to group A or B, but not group C or D, as genes with strong potential to be relevant to HCC-HCV (Supplementary Figure 3). In addition, 21 miRNA transcripts were observed to be differentially expressed in the developed HCC samples compared with the non-developed HCC samples in GSE119159, including nine upregulated and 12 downregulated transcripts.

### The Core Regulatory Module and Key Regulators

To mine HCV-HCC-related regulatory relationships, we first constructed a TF–miRNA–mRNA regulatory network as a background network. Then, the HCV-induced HCC-related subnetwork was constructed by mapping DEGs into the background network. The nodes in the subnetwork contained DEGs and genes directly connected to the DEGs. In total, 359 TFs, 395 miRNAs, 739 target genes, and 2626 edges were present in the subnetwork.

**FIGURE 3:**
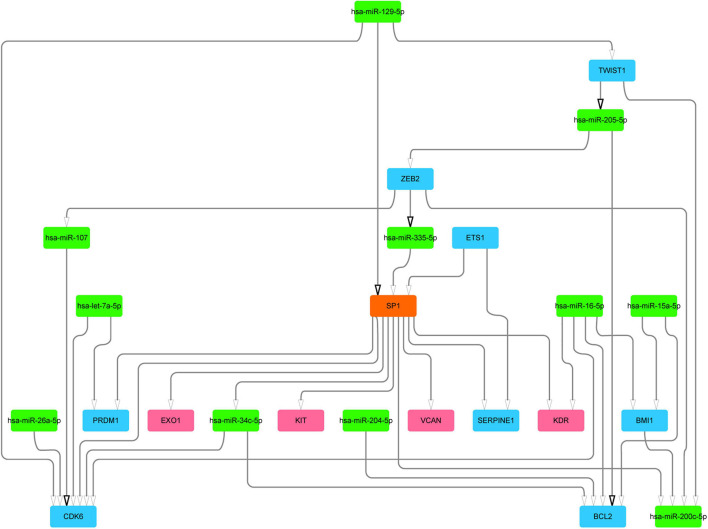
The core regulatory module and key regulators. Blue color represents DEGs, green color represents DE-miRs, and red color represents different mutation genes. DEGs, differentially expressed genes; DE-miRs, differentially expressed microRNAs.

We next mined the core regulatory module from the subnetwork by extracting the top 20 nodes ranked by closeness centrality and the edges among them. Notably, the regulatory relationships between these 20 nodes and differential mutated genes were added into the core regulatory module (Figure 3 and Supplementary Table 2). Finally, the core regulatory module contained 24 nodes and 36 edges.

**FIGURE 4:**
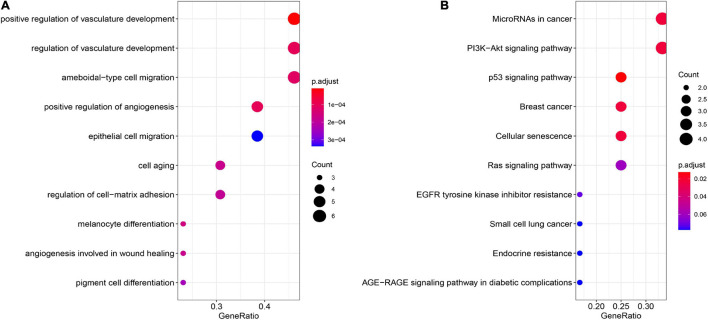
GO and KEGG pathway enrichment analyses. **(A)** GO enrichment analysis of the module genes. **(B)** KEGG pathway enrichment analysis of the module genes. GO, Gene Ontology; KEGG, Kyoto Encyclopedia of Genes and Genomes.

To analyze the function of genes in the module, we conducted enrichment analysis of GO and KEGG, with F*DR* < 0.05 used as the cutoff to identify statistically significant GO terms and KEGG pathways. We found that many GO terms and KEGG pathways were implicated in the HCV-HCC processes in previous studies. As shown in Figure 4A and Supplementary Table 3, in the biological process and molecular function categories, the significantly enriched genes were for vasculature development regulation (Vescovo et al., 2016), ameboidal-type and epithelial cell migration (Khera et al., 2017), cell aging (Naggie, 2017), cell-matrix adhesion (Ninio et al., 2019), and melanocyte differentiation and angiogenesis involved in wound healing (Mohsen et al., 2014). Furthermore, KEGG pathway analysis showed that the significantly enriched genes were for small cell lung cancer, miRNAs in cancer, PI3K–Akt signaling pathway (Cheng et al., 2015), Ras and p53 signaling pathways (Vescovo et al., 2016), cellular senescence (Shiu et al., 2017), endocrine resistance, and advanced glycation end products (AGE)–receptor for AGE (RAGE) signaling pathway in diabetic complications (Hyogo and Yamagishi, 2008; Figure 4B and Supplementary Table 4).

**FIGURE 5:**
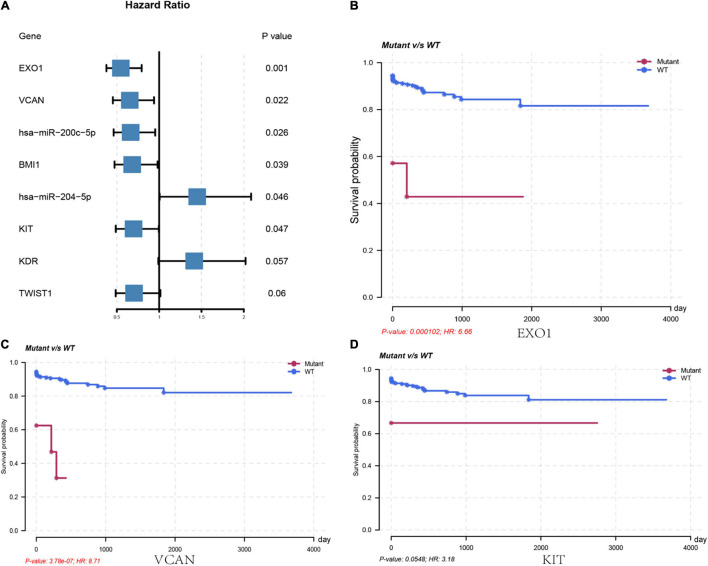
Key regulators are related to worse survival rate. **(A)** Forest plot of hazard ratios showing the prognostic values of genes. **(B–D)** Survival curves of key regulators in the LIHC patients from TCGA dataset. LIHC, liver hepatocellular carcinoma; TCGA, The Cancer Genome Atlas.

**FIGURE 6:**
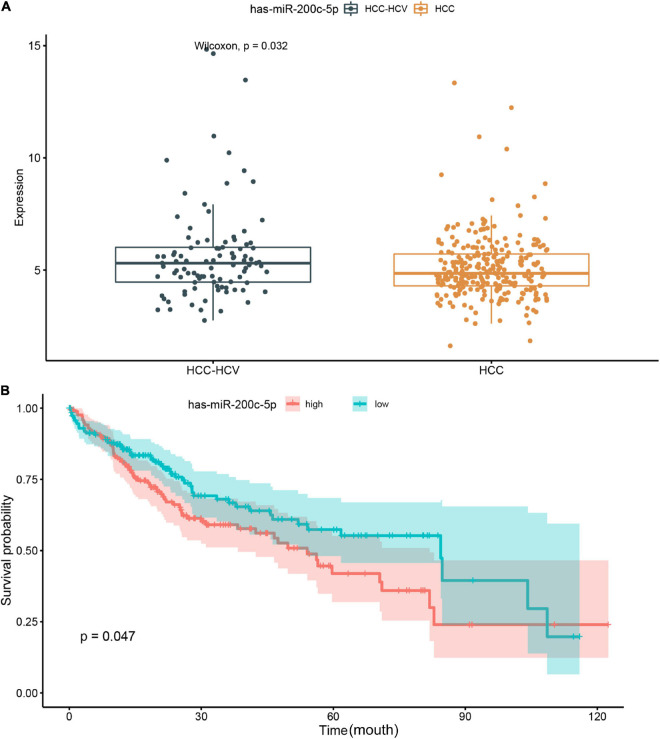
Relationship between miR-200c expression level and patient prognosis. **(A)** Significant difference was found in the miR-200c expression between HCC-HCV and HCC patients. **(B)** HCC patients with high miR-200c expression have a relatively poor prognosis. HCC, hepatocellular carcinoma; HCV, hepatitis C virus.

We further analyzed the genes in the core regulatory module and found that expression of *EXO1*, *VCAN*, *has-miR-200c-5p*, *BMI1*, *has-miR-204-5p*, and *KIT* was significantly correlated with HCC prognosis in all patients; and thus, these genes were considered key regulators (Figure 5A). In particular, we found that the patients with low *EXO1*, *VCAN*, or *KIT* expression had an adverse outcome (*HR* < 1; Figure 5A). The HCC-HCV patients with mutations in these three genes have possibly also poor prognoses (Figures 5B–D). They may be potential biomarkers to predict the prognosis of patients at the genomic level. Moreover, we found that *has-miR-200c-5P* was significantly overexpressed in HCC-HCV samples (Figure 6A). The survival time of patients with high *has-miR-200c-5P* expression was significantly lower than that of patients with low expression (Figure 6B), suggesting that *has-miR-200c-5P* may be a potential biomarker to predict the prognosis of patients at the expression level.

Mutations in specific locations in *EXO1* have been reported to inactivate proteins that increase cancer susceptibility (Welchew et al., 2002). *KDR* was also a significantly differential mutated gene in the module. KDR is the principal receptor that promotes the proangiogenic action of vascular endothelial growth factor and is involved in the tumorigenesis and progression of many malignancies, including HCC (Zheng et al., 2014). Moreover, *BCL2* was the downstream gene in the core regulatory module. *BCL2* can be functionally divided into antiapoptotic and proapoptotic groups. The balance between these two groups may determine the fate of a tumor cell. In HCC, this balance is often tilted toward the antiapoptotic members, leading to resistance to death and rapid proliferation in cancer cells (Alenzi et al., 2010). *BCL2* expression in the HCC-HCV samples was lower than that in the HCC samples, but the difference was non-significant in TCGA data—which may be a reason for the worse prognosis of HCC-HCV.

## Discussion

HCC is responsible for the second highest global mortality rate, and HCV infection is a leading HCC risk factor. However, the mechanisms of HCC initiation, development, and metastasis are too complicated and thus unclear (Kanda et al., 2019). Currently, several factors are believed to influence the evolution of HCC from HCV infection. However, due to the lack of appropriate models or data, determining the specific role of HCV in the malignant transformation of liver cells is difficult. To identify and characterize these mechanisms, researchers have conducted genomic, transcriptomic, and epigenomic studies (Khatun and Ray, 2019).

Driver mutations in cancer-associated genes alter downstream signaling and transcription patterns, which are critical in cancer progression (Lai and Yang, 2013; Zhang et al., 2014, 2015; Huh et al., 2019). These studies have revealed that downstream gene mutations and gene expression changes are critical in hepatitis-induced liver cancer development. In this study, we found that mutations in a single gene can have a significant impact on disease prognosis in patients, whereas a combination of mutations in multiple genes is not an effective predictor of prognosis. This may be due to the low probability of simultaneous mutations of multiple genes; this will be studied further in our future work. Genomic research has found that long-term interactions between hepatitis virus and immune system causes significant stress and damage to the liver cells, making them undergo pathological adaptation—even after elimination of the virus. Non-coding RNA (ncRNA)-related analysis has indicated that miRNAs play a crucial role in the posttranscriptional regulation of gene expression (Wong et al., 2018). Deregulation of certain miRNAs leads to the inactivation of tumor-suppressor genes and activation of HCC-related oncogenes. In this study, we incorporated whole-genome and transcriptomic sequence data to identify key regulators of HCV-HCC and found that abnormal expression of certain genes and miRNAs predict whether a patient with HCV infection will develop HCC. These genes may be potential biomarkers, which could enable HCC detection at significantly earlier stages.

In the functional enrichment analysis, we found that genes in the module were significantly enriched in the PI3K–Akt signaling pathway that promotes survival and growth in response to extracellular signals. KIT is an important receptor tyrosine kinase (RTK) that can stimulate the PI3K–Akt signaling pathway (Zhou et al., 2011). In addition, recent studies have shown that KIT exon 9 had a mutation resistant to TGFβ, which can promote HCC development in HCV patients (El-Houseini et al., 2019). The miR-200 family—the most common family of miRNAs—demonstrates low expression in various cancers and is closely associated with tumorigenesis and outcome, particularly in HCC (Mao et al., 2020). *has-miR-200c-5P* is significantly overexpressed in HCV patients and promotes hepatic fibrosis (Ramachandran et al., 2013)—consistent with our results. Moreover, the survival time of patients with high *has-miR-200c-5P* expression was significantly lower than that of patients with low expression in the current study. In general, *has-miR-200c-5P* overexpression in esophageal cancer increases resistance to chemotherapeutic drugs by dysregulating PI3K–Akt signaling pathway (Karakatsanis et al., 2013). Therefore, we speculate that *has-miR-200c-5P* and *KIT* may jointly regulate the PI3K–Akt signaling pathway and affect drug response and prognosis in HCV-HCC patients.

Although we identified some important regulatory genes and miRNAs, the specific underlying mechanisms could not be elaborated. Furthermore, HCC is complicated and multifactorial, and taking all factors into consideration was difficult. Therefore, additional studies determining whether genes correlated with HCV-induced cancer are also correlated with liver cancer caused by other factors are warranted.

## Data Availability Statement

The original contributions presented in the study are included in the article/Supplementary Material, further inquiries can be directed to the corresponding author/s.

## Author Contributions

GC: study design, manuscript writing, and data analysis. WZ: data analysis, data collection, and manuscript writing. YB: data analysis and data collection. All authors have read, edited and approved of the final version of the manuscript.

## Conflict of Interest

The authors declare that the research was conducted in the absence of any commercial or financial relationships that could be construed as a potential conflict of interest.

## Publisher’s Note

All claims expressed in this article are solely those of the authors and do not necessarily represent those of their affiliated organizations, or those of the publisher, the editors and the reviewers. Any product that may be evaluated in this article, or claim that may be made by its manufacturer, is not guaranteed or endorsed by the publisher.

## Footnotes

1
https://portal.gdc.cancer.gov/
