# Supplementary material for: Identification of Key Regulators of Hepatitis C Virus-Induced Hepatocellular Carcinoma by Integrating Whole-Genome and Transcriptome Sequencing Data
Source: Front Genet. 2021 Sep 9;12:741608. doi: 10.3389/fgene.2021.741608 (PMC8460086; doi:10.3389/fgene.2021.741608)
Supplement: Supplementary file 1 [file Data_Sheet_1.docx]

**Supplementary figure 1.** The odds ratio value of the highly mutated gene in the HCC-HCV samples


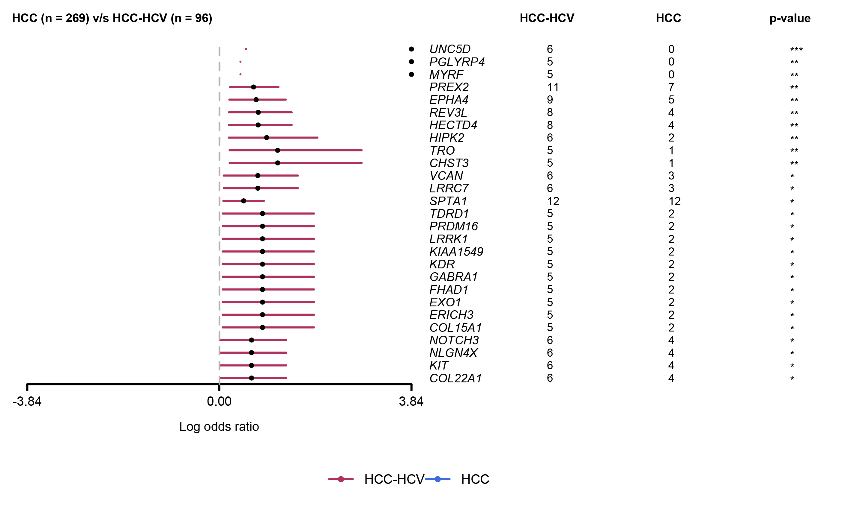


**Supplementary figure 2.** A: Clustering heat map of different genes grouped by the expression from the HCC-HCV and HCC samples. B: Volcano map of differentially expressed genes grouped by the expression from the HCC-HCV and HCC samples.


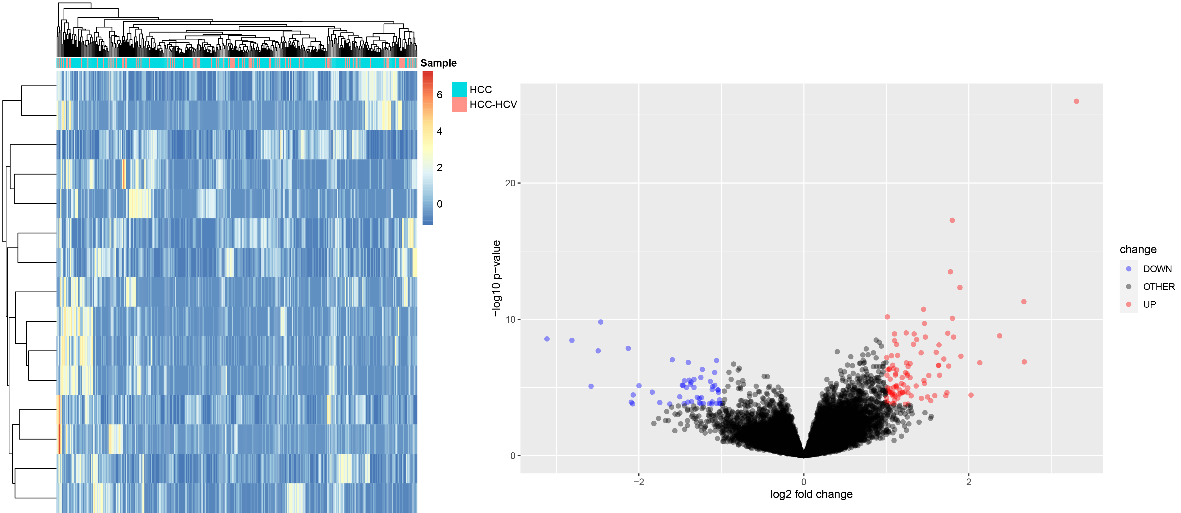


**Supplementary figure 3.** Venn diagram analysis of genes DEGs >2-fold (FDR < 0.05) between (A) HCC vs. HCC-HCV-adjacent, suggesting related to HCV-related carcinogenesis; (B) HCC-HCV vs. HCC, suggesting related HCV-related hepatocarcinogenesis; (C) HCC-HCV-adjacent vs. HCC-adjacent, suggesting related to HCV-related non-oncogenic effects, and (D) HCC vs. HCC-adjacent, suggesting related to non-HCV-related carcinogenesis.


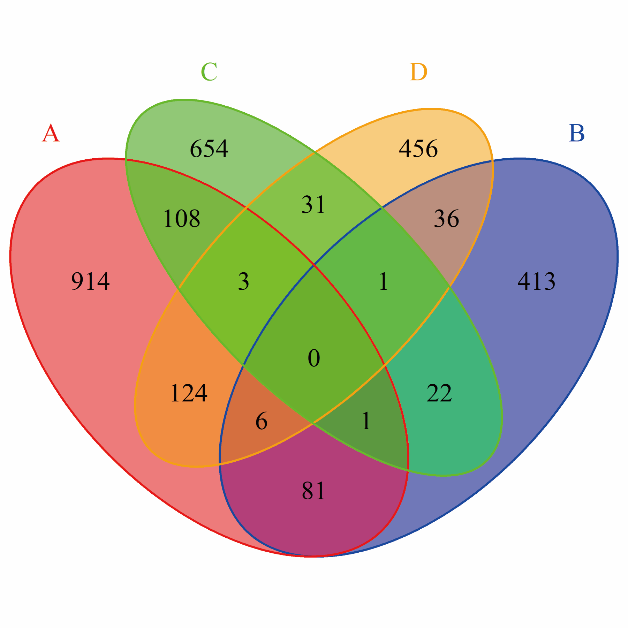


**Supplementary table 1.** 41 differentially mutated genes

| GENE NAME | HCC-HCV | HCC | p-value |
| --- | --- | --- | --- |
| UNC5D | 6 | 0 | 0.000294 |
| MYRF | 5 | 0 | 0.001164 |
| PGLYRP4 | 5 | 0 | 0.001164 |
| PREX2 | 11 | 7 | 0.001491 |
| EPHA4 | 9 | 5 | 0.002617 |
| HECTD4 | 8 | 4 | 0.003382 |
| REV3L | 8 | 4 | 0.003382 |
| HIPK2 | 6 | 2 | 0.005082 |
| CHST3 | 5 | 1 | 0.005512 |
| TRO | 5 | 1 | 0.005512 |
| LRRC7 | 6 | 3 | 0.011993 |
| VCAN | 6 | 3 | 0.011993 |
| SPTA1 | 12 | 12 | 0.013897 |
| COL15A1 | 5 | 2 | 0.01525 |
| ERICH3 | 5 | 2 | 0.01525 |
| EXO1 | 5 | 2 | 0.01525 |
| FHAD1 | 5 | 2 | 0.01525 |
| GABRA1 | 5 | 2 | 0.01525 |
| KDR | 5 | 2 | 0.01525 |
| KIAA1549 | 5 | 2 | 0.01525 |
| LRRK1 | 5 | 2 | 0.01525 |
| PRDM16 | 5 | 2 | 0.01525 |
| TDRD1 | 5 | 2 | 0.01525 |
| COL22A1 | 6 | 4 | 0.023612 |
| KIT | 6 | 4 | 0.023612 |
| NLGN4X | 6 | 4 | 0.023612 |
| NOTCH3 | 6 | 4 | 0.023612 |
| FAT3 | 11 | 12 | 0.025096 |
| MUC17 | 8 | 7 | 0.030309 |
| DNAH3 | 5 | 3 | 0.032195 |
| EPPK1 | 5 | 3 | 0.032195 |
| GRXCR1 | 5 | 3 | 0.032195 |
| MAB21L1 | 5 | 3 | 0.032195 |
| PCDH11X | 5 | 3 | 0.032195 |
| SORCS1 | 5 | 3 | 0.032195 |
| TMEM132D | 5 | 3 | 0.032195 |
| ZNF521 | 5 | 3 | 0.032195 |
| ABCB5 | 6 | 5 | 0.040959 |
| KIAA1217 | 6 | 5 | 0.040959 |
| ROBO2 | 6 | 5 | 0.040959 |
| SI | 6 | 5 | 0.040959 |
| ITPR3 | 7 | 6 | 0.046832 |

**Supplementary table 2.** Source of DEGs in the module

| GENE NAME | DEG in the TCGA | DEG in the GEO |
| --- | --- | --- |
| PRDM1 | - | Yes |
| CDK6 | - | Yes |
| ZEB2 | - | Yes |
| ETS1 | - | Yes |
| TWIST1 | Yes | - |
| SERPINE1 | - | Yes |
| BM1 | - | Yes |
| BCL2 | - | Yes |

**Supplementary table 3.** GO enrichment analysis of the module genes

| ONTOLOGY | ID | Description | FDR |
| --- | --- | --- | --- |
| BP | GO:1904018 | positive regulation of vasculature development | 6.74E-06 |
| BP | GO:0045766 | positive regulation of angiogenesis | 9.84E-05 |
| BP | GO:1901342 | regulation of vasculature development | 9.84E-05 |
| BP | GO:0001667 | ameboidal-type cell migration | 0.000118 |
| BP | GO:0030318 | melanocyte differentiation | 0.000159 |
| BP | GO:0007569 | cell aging | 0.000177 |
| BP | GO:0060055 | angiogenesis involved in wound healing | 0.000177 |
| BP | GO:0001952 | regulation of cell-matrix adhesion | 0.000189 |
| BP | GO:0050931 | pigment cell differentiation | 0.000241 |
| BP | GO:0010631 | epithelial cell migration | 0.000338 |

**Supplementary table 4.** KEGG pathway enrichment analysis of the module genes

| ID | Description | P value |
| --- | --- | --- |
| hsa04115 | p53 signaling pathway | 0.000145 |
| hsa05206 | MicroRNAs in cancer | 0.000813 |
| hsa05224 | Breast cancer | 0.001139 |
| hsa04151 | PI3K-Akt signaling pathway | 0.001338 |
| hsa04218 | Cellular senescence | 0.001353 |
| hsa04014 | Ras signaling pathway | 0.004202 |
| hsa01521 | EGFR tyrosine kinase inhibitor resistance | 0.005808 |
| hsa05222 | Small cell lung cancer | 0.007807 |
| hsa01522 | Endocrine resistance | 0.00882 |
| hsa04933 | AGE-RAGE signaling pathway in diabetic complications | 0.009171 |
